# Knowledge, attitude, and practice of artificial intelligence among doctors and medical students in Pakistan: A cross-sectional online survey

**DOI:** 10.1016/j.amsu.2022.103493

**Published:** 2022-03-14

**Authors:** Zaboor Ahmed, Khurram Khaliq Bhinder, Amna Tariq, Muhammad Junaid Tahir, Qasim Mehmood, Muhammad Saad Tabassum, Muna Malik, Sana Aslam, Muhammad Sohaib Asghar, Zohaib Yousaf

**Affiliations:** a Lahore General Hospital, Lahore, 54000, Pakistan; b Shifa International Hospital, Islamabad, Pakistan; c King Edward Medical University, Lahore, Pakistan; d Combined Military Hospital (CMH), Lahore, Pakistan; e Benazir Bhutto Hospital, Rawalpindi, Pakistan; f Dow University of Health Sciences–Ojha Campus, Karachi, Pakistan; g Hamad Medical Corporation, Doha, Qatar

**Keywords:** Artificial intelligence, Machine learning, Deep learning, Medical students, Curriculum

## Abstract

**Background:**

The use of Artificial intelligence (AI) has gained popularity during the last few decades and its use in medicine is increasing globally. Developing countries like Pakistan are lagging in the implementation of AI-based solutions in healthcare. There is a need to incorporate AI in the health system which may help not only in expediting diagnosis and management but also injudicious resource allocation.

**Objective:**

To determine the knowledge, attitude, and practice of AI among doctors and medical students in Pakistan.

**Materials and methods:**

We conducted a cross-sectional study using an online questionnaire-based survey regarding demographic details, knowledge, perception, and practice of AI. A sample of 470 individuals including doctors and medical students were selected using the convenient sampling technique. The chi-square test was applied for the comparison of variables.

**Results:**

Out of 470 individuals, 223(47.45%) were doctors and 247(52.55%) were medical students. Among these, 165(74%) doctors and 170(68.8%) medical students had a basic knowledge of AI but only 61(27.3%) doctors and 48(19.4%) students were aware of its medical applications. Regarding attitude, 237(76.7%) individuals supported AI's inclusion in curriculum, 368(78.3%) and 305(64.9%), 281(59.8%) and 269(57.2%) acknowledged its necessity in radiology, pathology, and COVID-19 pandemic respectively.

**Conclusion:**

The majority of doctors and medical students lack knowledge about AI and its applications, but had a positive view of AI in the field of medicine and were willing to adopt it.

## Highlights

• The majority of doctors and medical students lack knowledge about AI and its applications.
• Developing countries like Pakistan are lagging in the implementation of AI-based solutions in healthcare.

• There is a need to incorporate AI in the health system which may help in expediting diagnosis, management and injudicious resource allocation.
• More resources need to be allocated for the planning and implementation of AI in the medical curriculum.

## Abbreviations

AI: Artificial Intelligence
ML: Machine Learning
DL: Deep Learning
AMDC: Ameer-ud-din Medical College
ANMC: Azra Naheed Medical College
AIMC: Allama Iqbal Medical College
SIMS: Services Institute of Medical Sciences
GMC: Gujranwala Medical College
LMDC: Lahore Medical and Dental College
NSMC: Nawaz Sharif Medical College
SKZMDC: Sheikh Khalifa Bin Zayed Al Nahyan Medical and Dental College
RMU: Rawalpindi Medical University
PMU: Punjab Medical University
KSMC: Khawaja Safdar Medical College
KEMU: King Edward Medical University
FJMU: Fatima Jinnah Medical University
AJKMC: Azad Jammu Kashmir Medical College
QAMC: Quaid e Azam Medical College
SMDC: Shalamar Medical and Dental College
WMC: Wah Medical College
RMDC: Rahbar Medical College
RMI: Rehman Medical Institute
LGH: Lahore General Hospital
KMDC: Karachi Medical and Dental College
JPMC: Jinnah Post Graduate Medical Centre
FMH: Fatima Memorial Hospital

## 1 Introduction

Over the past few decades, artificial intelligence (AI) has gained unprecedented attention and is being called the fourth industrial revolution [[1], [2], [3]]. Developed countries have dedicated considerable resources for AI research and its implementation in health care [4]. Despite government policies for the promotion of AI, developing countries like Pakistan are still lagging in education, research, and implementation of AI in general and in healthcare in particular [1]. The existing lack of resources in developing countries is compounded by the pandemic and knowledge of AI and practical implementation in healthcare is essential for reducing workload and diagnostic errors [3,5].

Machine learning and deep learning are two subsets of AI that are being explored globally in the health sector [6]. The largest application of AI algorithms is witnessed in radiology but examples of its applications in other fields like dermatology, ophthalmology, psychiatry, cardiology, oncology, neurosciences, pathology and medicine are also available [2,[7], [8], [9], [10], [11], [12]]. AI algorithms aid radiologists in the detection of abnormal phenotypic characteristics in images, categorization, formulation of hypotheses regarding the underlying condition of the patient, type of procedure, and interpretation of results [13,14]. Inclusion of AI in pathology improves the predictive and prognostic properties of the existing methods of experimentation and laboratory testing and also improves the analysis of tissue histology and molecular data [7]. Studies have shown similar attributes of AI in dermatology in which it provides robustness in diagnostic imaging and assessment of numerous benign and malignant dermatological pathologies and in ophthalmology where it aids in diagnosis and evaluation of multiple retinal and other ophthalmic abnormalities [[15], [16], [17], [18]]. AI subsets of machine learning and deep learning also plays role in the medical education of undergraduate medical students and trainees in post-graduate programs [19]. AI program has been introduced under the presidential initiative in Pakistan, but there are multiple challenges for the implementation of AI in the health sector including provider's inertia, financial restrictions, limitation of trained health professionals for setting up diagnostic protocols on which algorithms are based, lack of data on public perception and implications of AI, and fear of replacement of physician, social barriers, confidentiality, and medicolegal implications [1,8,19].

The purpose of this research is to determine the extent of knowledge and perception of doctors and medical students of Pakistan regarding AI and its implications and assess the knowledge of current practices of AI in Pakistan. It is hypothesized that medical students are not fully aware of the AI implications in medical sciences.

## 2 Methodology

### 2.1 Study design and sample size

This study was conducted using an online questionnaire-based cross-sectional open survey from 1st May 2021 to 5th June 2021. The questionnaire was prepared using Google docs and the questionnaire forms were sent to medical students and doctors friend circle forwarding and social media apps like WhatsApp, Facebook, and Messenger. Questionnaire was developed by two independent investigators and any discrepancy was resolved by discussion. The only medical students’ groups were targeted on social media for dissemination. The questionnaire was checked and validated by a senior faculty member. No personal information was collected or stored, and access to data was authorized to primary investigator only. Duration of minimum 1 month was selected for this process and two to three times reminders were given. A total of 470 individuals responded to the survey including doctors of different healthcare centers and medical students of different medical and dental colleges of Pakistan. The sample population was selected using a convenience sampling technique and was questioned regarding their demographic details (age, gender, level of qualification, affiliated institute), knowledge about AI and its applications, perception of AI and its applications and practices of AI. A pilot survey was conducted before fielding the questionnaire on a limited number of responders to assess the usability and technical functionality of the online questionnaire. Respondents were able to review and change their answers. Duplicate entries were removed from the analysis., and only completed questionnaires were included in the analysis. Inclusion criteria were responders being medical personnel and completed survey. Exclusion criteria were non-medical responders and incomplete survey.

### 2.2 Ethical approval

The study was an observational study that valued the anonymity and autonomy of the participant. Participants were allowed to withhold the completed form from the submission. The study did not contain any names or emails so that the participant could not be tracked. The study ensured that the privacy of each participant was adequately protected. Ethical approval was obtained from the Research Ethical Committee having approval number: 80/16/21. The study was conducted in full conformance with principles of the Declaration of Helsinki. Informed consent was obtained from all the participants before filling the survey.

### 2.3 Statistical analysis

SPSS version 25.0 was used to analyze the data. Frequency tables were used to plot the frequencies of variables. A Chi-square test was applied to determine the statistical association between the variables with a p-value of <0.05 was considered significant. Univariate logistic regression was also applied to obtain unadjusted odds ratio and their 95% confidence intervals.

## 3 Results

Out of 470 (100%) individuals, there were 247(52.6%) undergraduates at different medical colleges of Pakistan and 223(47.45%) were doctors with different professional statuses in different institutes of Pakistan. The baseline characteristics of all populations are given in Table 1. Among undergraduates, the majority belonged to Ameer-ud-din medical college, Lahore. Out of 223 doctors, 64(13.6%) were postgraduate residents, 29(45%) of which were affiliated with Lahore General Hospital (LGH), Lahore, 13(20.3%) had an affiliation with Punjab Dental Hospital, Lahore, and 4(6.24%) had an affiliation with Shifa International Hospital, Islamabad. Only 2(3.1% each) postgraduate residents were from CMH Abottabad, Mayo Hospital Lahore, and Pakistan Institute of Neurosciences, Lahore, and only 1(1.6% each) postgraduate resident was from ANMC, FMH, Chughtai institute of Pathology, Sheikh Zayd hospital (Rahim Yar Khan), RMI (Peshawar), Nishtar Hospital (Multan), Chaudhary M. Akram Hospital, Civil Hospital Karachi, Khyber Medical University (Peshawar), JPMC (Karachi), social security hospital (Lahore) and DHQ Gujranwala each.

**Table 1:** Baseline Characteristics of the study population.

Sr. no	Variable	Value
1	Age	
	• 10-20	• 60(12.8%)
	• 21-30	• 386(82.1%)
	• 31-40	• 20(4.3%)
	• 41-50	• 2(0.4%)
	• 51-60	• 2(0.4%)
2	Gender	
	• Male	• 239(50.9%)
	• Female	• 231(49.1%)
3	Qualification level:	
	• Graduate/Postgraduate	• 223(47.4%)
	• Under Graduate	• 247(52.6%)
4	Graduate/Postgraduate	223(47.45%)
	• House Officer	• 54(24.2%)
	• Senior House Officer	• 18(8.1%)
	• Medical Practitioner	• 19(8.5%)
	• Medical Officer	• 57(25.6%)
	• Post Graduate Resident	• 64(28.7%)
	• Senior Registrar	• 4(1.8%)
	• Assistant Professor	• 4(1.8%)
	• Associate Professor	• 1(0.45%)
	• Professor	• 2(0.9%)
5	Under Graduate/Medical Students	247(52.6%)
	• 1st professional	• 40(16.2%)
	• 2nd professional	• 33(13.4%)
	• 3rd professional	• 37(15%)
	• 4th professional	• 49(19.8%)
	• 5th professional	• 88(35.6%)
6	No. of undergraduates in medical colleges	247(52.6%)
	• AMDC, Lahore	• 150 (60.7%)
	• AIMC, Lahore	• 20 (8.1%)
	• SIMS, Lahore	• 17(6.9%)
	• GMC, Gujranwala	• 15(6.1%)
	• ANMC, Lahore	• 9(3.6%)
	• NSMC, Gujrat	• 6(2.4%)
	• AJKMC, Muzaffarabad	• 7(2.8%)
	• QAMC, Bahawalpur	• 4(1.6%)
	• KEMU, Lahore	• 3(1.2%)
	• SKZMDC, Lahore	• 3(1.2%)
	• PMU, Faisalabad	• 3(1.2%)
	• KSMC, Sialkot	• 2(0.8%)
	• CPMC, Lahore	• 1(0.4%)
	• FJMU, Lahore	• 1(0.4%)
	• KMDC, Karachi	• 1(0.4%)
	• WMC, Rawalpindi	• 1(0.4%)
	• RMDC, Lahore	• 1(0.4%)
	• RMU, Rawalpindi	• 1(0.4%)
	• SMDC, Lahore	• 1(0.4%)
	• LMDC, Lahore	• 1(0.4%)

### 3.1 Knowledge of AI

Regarding knowledge of AI, individuals were questioned about the basic concept of AI, its subtypes i.e., (machine learning (ML) and deep learning ML and DL(DL), and its applications. It was observed that 335(71.28%) had a basic concept about AI but only 166(35.3%) had knowledge about ML and DL and only 109(23.2%) had knowledge about its applications. 135(28.7%) individuals had no knowledge about the basic concept of AI, 304(64.7%) had no knowledge about ML and DL, and 361(76.8%) were unaware of any application of AI in the medical field. Only 116(24.7%) individuals were aware of the application of AI in radiology and only 88(18.7%) knew AI application in pathology. Few of the applications of AI known to the individuals were in Robotic Surgery, Diagnostic Radiology, Crispr technology, Diagnostic imaging in ophthalmic pathologies, 3D Anatomical study, Risk assessment in cardiac patients by imaging techniques, Automated ventilators, radiological imaging modalities like MRI, CT scan, X rays and ultrasound, stroke assessment, radiotherapy in cancer patients, histological imaging in pathology laboratories and electrocardiogram (ECG) assessment for cardiac anomalies. The correlation of Knowledge of AI with different variables with Odds ratios is given in Table 2. It was observed that lack of curriculum at the undergraduate level and during postgraduate residency training and gender were significant factors affecting the knowledge of AI with P values of less than 0.05. Males were found to have more knowledge about AI than females. The qualification level was not a significant factor for the knowledge of AI with a P-value greater than 0.05.

**Table 2:** Correlation of Knowledge, Attitude and Practice of Artificial intelligence with different variables.

Factors	Been taught about AI in medical school/PG training, n (%)	Not being taught in medical school/PG training, n (%)		Odds ratio	P value
1. Know about AI				–	<0.001
Yes	33(9.9%)	302 (91.9%)			
No	0(0.0%)	135 (100.0%)			
2. Know about ML and DL				4.069 (1.912–8.621)	<0.001
Yes	11(13.3%)	144(86.7%)			
No	22(3.6%)	293(96.4%)			
3. Know about any application in medicine				2.309 (1.108–4.811)	0.022
Yes	13(11.9%)	96(88.1%)			
No	20(4.5%)	341(94.5%)			
4. AI is essential in the field of medicine				–	<0.001
Strongly agree	17(13.2%)	112(86.8%)			
Agree	11(5.0%)	210(95.0%)			
Disagree	1(10.0%)	9(90.0%)			
Strongly disagree	2(40.0%)	3(60.0%)			
No opinion	2(98.1%)	103(1.9%)			
5. Applied AI				10.175 (4.753–21.784)	<0.001
Yes	16(30.2%)	37(69.8%)			
No	17(4.1%)	400(95.9%)			
**Qualification level**					
**Factors**	**Undergraduate, n (%)**		**Graduate/Post graduate, n (%)**	**Odds ratio**	**P value**
1. Know about AI				1.289 (0.862–1.927)	0.217
Yes	170(49.3%)		165(50.7%)		
No	77(57.0%)		58(43.0%)		
2. Know about ML and DL				1.360 (0.931–1.988)	0.111
Yes	79(16.8%)		87(18.5%)		
No	168(55.3%)		136(44.7%)		
3. Know about any application in medicine				1.561 (1.014–2.403)	0.042
Yes	48(44.0%)		61(56.0%)		
No	199(55.1%)		162(44.9%)		
4. AI is essential in the field of medicine				–	0.054
Strongly agree	65(50.4%)		64(49.6%)		
Agree	112(50.7%)		109(49.3%)		
Disagree	3(30.0%)		7(70.0%)		
Strongly disagree	1(20.0%)		4(80.0%)		
No opinion	66(62.9%)		39(37.1%)		
5. Applied AI				1.971 (1.095–3.549)	0.101
Yes	20(37.7%)		33(62.3%)		
No	227(54.4%)		190(45.6%)		
**Gender**					
**Factors**	**Male n (%)**		**Female n (%)**	**Odds ratio**	**P value**
1.Know about AI				1.680 (1.120–2.521) –	0.012
Yes	177(52.8%)		158(47.2%)		
No	54(40.0%)		81(60.0%)		
2. Know about ML and DL				1.476 (1.009–2.159)	0.044
Yes	92(55.4%)		74(44.6%)		
No	139(45.7%)		165(54.3%)		
3.Know about any application in medicine				1.916 (1.237–2.969)	0.003
Yes	67(61.5%)		42(38.5%)		
No	164(45.4%)		197(54.6%)		
4. AI is essential in the field of medicine				–	0.170
Strongly agree	72(55.8%)		57(44.2%)		
Agree	111(50.2%)		110(49.8%)		
Disagree	4(40.0%)		6(60.0%)		
Strongly disagree	2(40.0%)		3(60.0%)		
No opinion	42(40.0%)		63(60.0%)		
5. Applied AI				1.825 (1.014–3.285)	0.043
Yes	33(62.3%)		20(37.7%)		
No	198(475%)		219(52.5%)		

AI: artificial intelligence; ML: machine learning; and DL: deep learning.

### 3.2 Attitude towards AI

**Fig. 1:**
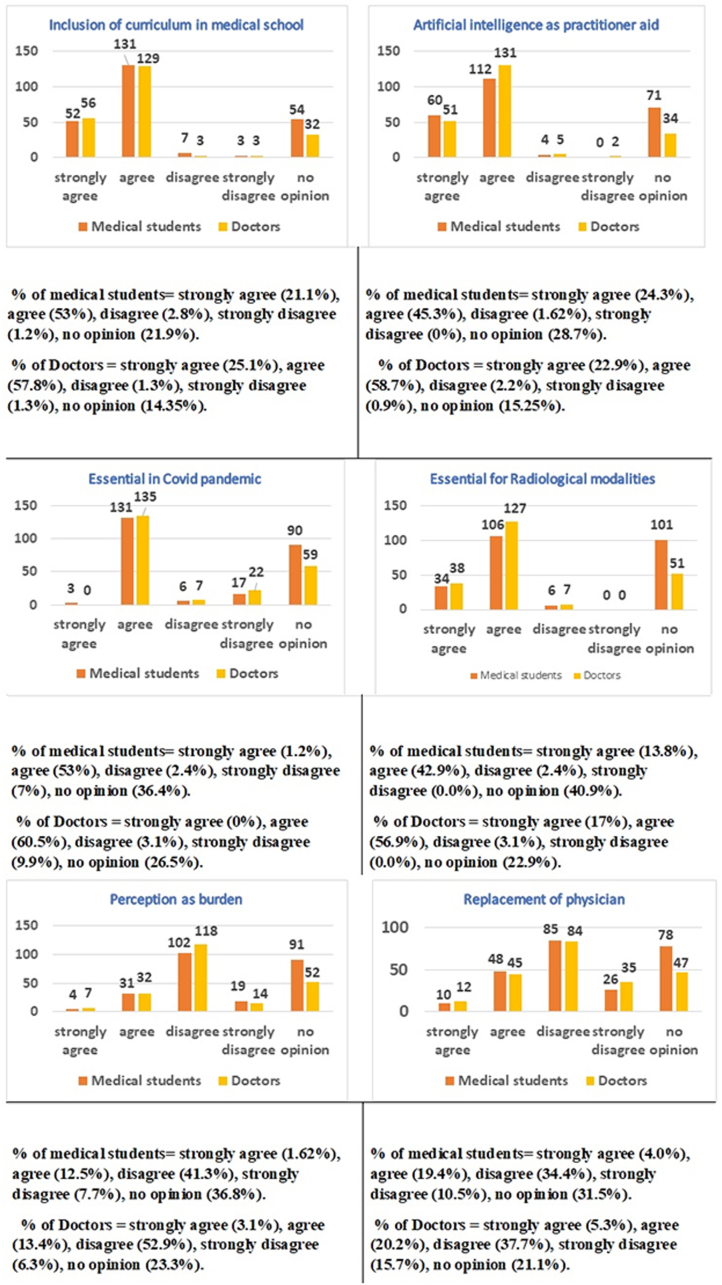
Perception towards artificial intelligence and its applications.

**Fig. 2:**
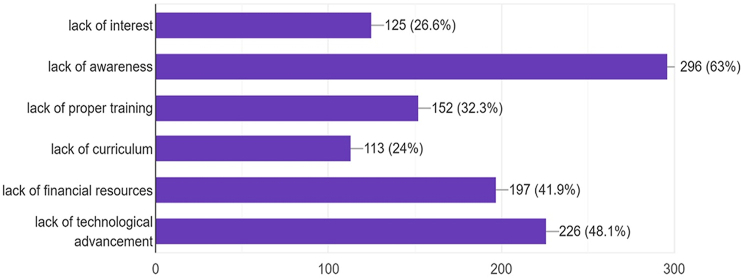
Causes of failure of implementation of AI in Pakistan: opinion of doctors and medical students.

Concerning the attitude towards AI in the health sector, 129(27.4%) individuals strongly agree and 221(47%) agree that AI is essential in the medical field while only 5(1.1%) strongly disagree and only 10(2.1%) disagree with this. About 105(22.3%) had no opinion regarding this and among them, the majority were females. Also, 8(3.2%) medical students and 14(6.3%) doctors strongly agreed and 67(27.1%) medical students and 67(30%) doctors agreed that AI implementation will reduce the errors in diagnosis while 6(2.4%) medical students and 9(4%) doctors strongly disagreed and 69(27.9%) medical students and 75(33.6%) doctors disagreed regarding the contribution of AI in diagnostic error reduction. The rest of the 155(32.9%) individuals had no opinion. 27(10.9%) medical students and 27(12.1%) doctors strongly agreed and 110(44.5%) medical students and 117(52.5%) doctors agreed that AI is essential in pathological diagnostic techniques. Meanwhile, A total of 164(34.9%) individuals had no opinion. Fig. 1 shows the attitude of doctors and medical students towards AI application in radiology and its need in the COVID-19 pandemic, the inclusion of curriculum in medical education and residency training, the role of AI as practitioner's aid, and fear of AI as a burden and a replacement of physician. According to the opinion of doctors and medical students, the causes of the failure of AI implementation in Pakistan are shown in Fig. 2. A total of 229(48.7%) individuals believe that an appropriate budget should be allocated for the promotion and implementation of AI in the health sector, 107(22.7%) disagreed with this notion and 126(26.8%) gave no opinion about it. Correlation of attitude towards AI essentialness in the medical field with variables like gender, lack of curriculum, and qualification level along with Odds ratio is given in Table 2 which shows that the lack of curriculum is a significant factor with a P-value of <0.05 and gender has no significant effect on attitude.

### 3.3 Practices of AI

Only 53(11.3%) including 20(8.1%) medical students and 33(14.8%) doctors had ever practically applied AI and all agreed that it made their respective tasks easy to complete. Meanwhile, the rest of the 417(88.7%) individuals including 227(91.9%) medical students and 190(85.2%) doctors had never applied AI in any task. 40(8.5%) had practically applied AI in radiology using modalities of X-ray, CT scan, and MRI for diagnostic and research purposes. 32(6.8%) individuals had experience of the practical application of AI in pathology for culture and sensitivity and histopathological testing for diagnosis and research purposes. Concerning the practice in the future, 318(67.7%) individuals including 154(62.3%) medical students and 164(73.5%) doctors were ready to practically apply AI in the future, and 86(34.8%) medical students and 52(23.3%) doctors did not have any opinion-whether or not they would work with AI in future. Correlation of current practice of AI with different variables along with Odds ratio is given in Table 2 which shows that lack of curriculum and gender are significant factors affecting the practice of AI with p values < 0.05.

## 4 Discussion

AI has revolutionized healthcare delivery [5], as it allows the tasks to be completed efficiently and accurately by using algorithms based on human intelligence [20]. Machine learning is a subtype of AI which is based on algorithms that require pre-calculated data and feature input while deep learning is more advanced in this regard that it skips the need for pre-designed classification and features [3,20]. During the recent COVID-19 pandemic, healthcare facilities and healthcare workers faced a crisis due to the reallocation of resources. In developing countries like Pakistan, there is an urgent need for AI tools that are patient-centered and assist physicians in diagnosis and treatment [8,21].

Pakistan is still in the initial phases of AI introduction and implementation with little native data available. Our research was aimed at the population of medical students and doctors of Pakistan concerning the different aspects of knowledge, attitude, and practice of AI in the field of medicine. A total of 470 individuals (medical students and doctors) participated in the study, out of which 50.9% were males and 49.1% were females with a male to female ratio of 1.03. Of 470 participants, 71.3% had a basic knowledge of AI but only 35.3% knew about its subtypes; ML and DL. Most of the individuals knowing AI were males and almost 77% of the study participants weren't aware of the practical application of AI in medicine. This shows that Pakistani doctors and medical students, despite having the basic knowledge of AI, don't know its practical implications.

Three-fourth (74.4%) of the study population acknowledged the importance of AI in modern diagnostics and considered it essential in advanced medicine. This is consistent with the findings of a study conducted in medical institutes of the UK in which a three-fourth majority of the students acknowledged the essential role of AI in the field of medicine [9]. Another related research was conducted on 98 health professionals of NHS trust, London which concluded that more than two-fifth had no understanding of ML and DL and 79% considered it essential in healthcare which is corresponding to our results [22].

In our study, 66.6% participants agreed on the notion that implementation of AI in medicine will reduce diagnostic errors which is consistent with the findings of a study done in three premier medical colleges of India concluding that 89% of the students expressed optimistic views regarding the implementation of AI in healthcare [23]. Also, 69.6% of medical students and 81.8% of doctors from the study population acknowledged that AI can serve as a practitioner's aid soon and most of them don't consider AI as a physician's replacement but rather a physician's diagnostic aid. The majority of the individuals (74% students and 83% doctors) also agreed on the inclusion of curriculum of AI in medical schools which is similar to study results from researches conducted in the USA [24] and Pakistan [1] in which the majority of students and doctors considered AI essential for the field of medicine and agreed over its inclusion in the curriculum.

Major causes of failure of implementation of AI in Pakistan as expressed by study participants include lack of adequate knowledge and awareness, disinterest in the field, poor training, no curriculum, low financial resources, and lack of technological advancements in our country. Furthermore, the majority of the study participants (56.7% students and 74% doctors) considered AI essential in advanced radiology and most of them agreed on its importance in the COVID-19 pandemic due to the reallocation of healthcare resources.

To summarize, our results showed that the majority of the doctors and medical students had basic knowledge about AI but lacked detailed knowledge about its applications in the medical field. Overall, the attitude of doctors and medical students towards the need for AI in the medical field was satisfactory and the majority considered it essential in radiology, pathology, and other fields of medicine. Most of the individuals responded in agreement with the idea of the inclusion of AI curriculum in medical colleges and postgraduate residency training and considered it a physician's aid in early diagnosis as well as in error reduction instead of a physician replacement. It was also observed that only a minority of individuals (11.3%) had ever experienced the practical application of AI in the medical field for diagnostic and research purposes which was in radiology based on modalities of X-ray, CT scan, and MRI and in pathology based on histopathological tests and culture and sensitivity testing. Our research is unique in that it is one of the first that gives insight into the extent of knowledge, attitude, and practice of students and doctors working in different institutes of Pakistan along with the factors affecting these. Since there is a need to address the willingness to adapt innovation and bring awareness of AI applications in current Medicine, it is recommended to design and implement an appropriate AI curriculum in the medical field in Pakistan as AI will play a progressively larger and more important role in the future of medicine and healthcare. The senior decision makers should aim developing policies to bring about the innovations in the field.

## 5 Limitations

Our research provides the basic understanding required in the inclusion of AI curriculum in developing countries like Pakistan. Our study has several limitations. Firstly, due to COVID-19 pandemic the distribution of the questionnaire was carried out online and not physically, which may precipitate a selection bias, which could have affected our results otherwise. Secondly, the sample size was small. Also, recall bias cannot be ignored. Using a convenience sample may have significant limitations in terms of its generalizability to the population. Since the questionnaire was tested on a convenience basis and biases the results to younger people. The age groups over 40 years are not represented enough.

## 6 Conclusion

The majority of doctors and medical students lack detailed knowledge about AI and its applications in healthcare, but had a positive view about it and were willing to practically adopt it. More resources need to be allocated for the planning and implementation of AI in the medical curriculum and for training the doctors to apply AI in their daily practice. Further research should be done to delve more into the attitude of individuals regarding the significance of artificial intelligence in the modern world.

## Ethics approval

Ethical approval was taken from research committee of Lahore General Hospital (approval no: 80/16/21).

## Funding

None.

## Author's contributions

K.K.B, Z.A, and M.M conceived the idea; K.K.B, Z.A, A.T, M.S.T, and S.A, collected the data; Z.A and M.S.A analyzed and interpreted the data; Z.A, M.J.T, Q.M, K.K.B, M.S.T, S.A, and Z.Y did write up of the manuscript; and finally, Z.Y, M.S.A, M.J.T, and M.M reviewed and revised the manuscript for intellectual content critically. All authors approved the final version of the manuscript.

## Registration of research studies

Name of the registry: Ethical Review Committee of Lahore General Hospital.
Unique Identifying number or registration ID: (Approval no: 80/16/21)
Hyperlink to your specific registration (must be publicly accessible and will be checked):

## Guarantor

Muhammad Sohaib Asghar and Muhammad Junaid Tahir.

## Consent

Consent to participate was taken from all the participants via online platform.

## Provenance and peer review

Externally peer reviewed, not commissioned.

## Declaration of competing interest

None.

## References

[1] Abid S., Awan B., Ismail T. (2019). Artificial intelligence: medical student s attitude in district Peshawar Pakistan. Pak. J. Public. Health..

[2] Waymel Q., Badr S., Demondion X., Cotton A., Jacques T. (2019). Impact of the rise of artificial intelligence in radiology: what do radiologists think?. Diagn. Interv. Imaging.

[3] Ooi S.K., Makmur A., Soon A.Y. (2021). Attitudes toward artificial intelligence in radiology with learner needs assessment within radiology residency programmes: a national multi-programme survey. Singapore. Med. J..

[4] Wahl B., Cossy-Gantner A., Germann S., Schwalbe N.R. (2018). Artificial intelligence (AI) and global health: how can AI contribute to health in resource-poor settings?. BMJ. Glob. Health..

[5] Xiang Y., Zhao L., Liu Z. (2020). Implementation of artificial intelligence in medicine: status analysis and development suggestions. Artif. Intell. Med..

[6] Shinners L., Aggar C., Grace S., Smith S S. (2021). Exploring healthcare professionals' perceptions of artificial intelligence: validating a questionnaire using the e-Delphi method. Digit. Health..

[7] Sarwar S., Dent A., Faust K., Richer M., Djuric U., Van Ommeren R., Diamandis P. (2019). Physician perspectives on integration of artificial intelligence into diagnostic pathology. NPJ. Digit. Med..

[8] Guo J., Li B. (2018). The application of medical artificial intelligence technology in rural areas of developing countries. Health. Equity..

[9] Sit C., Srinivasan R., Amlani A., Muthuswamy K., Azam A., Monzon L., Poon D.S. (2020). Attitudes and perceptions of UK medical students towards artificial intelligence and radiology: a multicentre survey. Insights. Imaging.

[10] Yan Y., Zhang J.W., Zang G.Y., Pu J. (2019). The primary use of artificial intelligence in cardiovascular diseases: what kind of potential role does artificial intelligence play in future medicine?. J. Geriatr. Cardiol..

[11] Shimizu H., Nakayama K.I. (2020). Artificial intelligence in oncology. Cancer Sci..

[12] Hassabis D., Kumaran D., Summerfield C., Botvinick M. (2017). Neuroscience-inspired artificial intelligence. Neuron.

[13] Hosny A., Parmar C., Quackenbush J., Schwartz L.H., Aerts H.J. (2018). Artificial intelligence in radiology. Nat. Rev. Cancer.

[14] Kahn C.E. (1994). Artificial intelligence in radiology: decision support systems. Radiographics.

[15] Li C.X., Shen C.B., Xue K. (2019). Artificial intelligence in dermatology: past, present, and future. Chin. Med. J..

[16] Du‐Harpur X., Watt F.M., Luscombe N.M., Lynch M.D. (2020). What is AI? Applications of artificial intelligence to dermatology. Br. J. Dermatol..

[17] Ting D.S., Pasquale L.R., Peng L. (2019). Artificial intelligence and deep learning in ophthalmology. Br. J. Ophthalmol..

[18] Moraru A.D., Costin D., Moraru R.L., Branisteanu D.C. (2020). Artificial intelligence and deep learning in ophthalmology-present and future. Exp. Ther. Med..

[19] Chan K.S., Zary N. (2019). Applications and challenges of implementing artificial intelligence in medical education: integrative review. JMIR. Med. Edu..

[20] Laï M.C., Brian M., Mamzer M.F. (2020). Perceptions of artificial intelligence in healthcare: findings from a qualitative survey study among actors in France. J. Transl. Med..

[21] Rodela T.T., Tasnim S., Mazumder H., Faizah F., Sultana A., Hossain M.M. (2020).

[22] Castagno S., Khalifa M. (2020). Perceptions of artificial intelligence among healthcare staff: a qualitative survey study. Front. Artif. Intell..

[23] Jindal A., Bansal M. (2020). Knowledge and education about artificial intelligence among medical students from teaching institutions of India: a brief survey. MedEdPublish.

[24] Wood E.A., Ange B.L., Miller D.D. (2021). Are we ready to integrate artificial intelligence literacy into medical school curriculum: students and faculty survey. J. Med. Educ. Curric. Dev..

